# Dielectric properties of Y and Nb co-doped TiO_2_ ceramics

**DOI:** 10.1038/s41598-017-09141-0

**Published:** 2017-08-17

**Authors:** Xianwei Wang, Bihui Zhang, Linhai Xu, Xiaoer Wang, Yanchun Hu, Gaohang Shen, Lingyun Sun

**Affiliations:** 1 Laboratory of Functional Materials, College of Physics and Materials Science, Henan Normal University, Xinxiang, 453007 China; 2 Henan Key Laboratory of Photovoltaic Materials, Xinxiang, 453007 China

## Abstract

In this work, the (Y_0.5_Nb_0.5_)_x_Ti_1−x_O_2_ (x = 0.001, 0.01, 0.02, 0.04, 0.06 and 0.1) ceramics (as called YNTO) were fabricated by synthesized through a standard solid-state reaction. As revealed by the X-ray diffraction (XRD) spectra, the YNTOs exhibit tetragonal rutile structure. Meanwhile, the grain size of YNTO ceramics increased and then decreased with the increase of x value, and the largest value reached when x = 0.02. All the YNTO samples display colossal permittivity (~10^2^–10^5^) over a wide temperature and frequency range. Moreover, the optimal ceramic, (Y_0.5_Nb_0.5_)_0.02_Ti_0.98_O_2_, exhibits high performance over a broad temperature range from 20 °C to 180 °C; specifically, at 1 kHz, the dielectric constant and dielectric loss are 6.55 × 10^4^ and 0.22 at room temperature, and they are 1.03 × 10^5^ and 0.11 at 180 °C, respectively.

## Introduction

Dielectric materials with colossal permittivity (CP) attract vast research interests due to their great potential applications in wide fields such as device miniaturization and energy storage^1–4^. As typical CP materials, the systems of BaTiO_3_, CaCu_3_Ti_4_O_12_ (CCTO), and doped-NiO have been widely explored^5–12^. Unfortunately, BaTiO_3_ ceramics only show colossal permittivity in a narrow temperature range which is even close to the temperature for phase transition (from ferroelectric to paraelectric phase). Furthermore, the reported work verified that CCTO ceramics display poor stability of their dielectric properties when they were subjected to frequency and temperature variations. Worse still, large dielectric loss and high variation of dielectric constant with temperature impeded the practical application of NiO-based system^6–9, 11^. Therefore, huge efforts need to be input in searching for new CP materials which can maintain high dielectric constant and low dielectric loss under a wide range of temperature and frequency.

Previous researches proposed AB co-doped rutile TiO_2_ ceramics (A was introduced as an electron-acceptor, such as In^3+^; B was introduced as an electron-donor, such as Nb^5+^) as a new sequence of CP materials^1, 2, 4, 13, 14^. The existence of a giant dielectric constant (ε_r_ ~ 6 × 10^4^) along with a low dielectric loss (tan δ < 0.02) at room temperature over varied frequency from 10^2^ to 10^6^ Hz was found in their research^1^. In fact, Hu *et al*. have illustrated that the In-doping lower the tan δ while Nb ions enhance the ε_r_, respectively. The localization of the hopping electrons was found to be near the designated lattice defect states which generate giant defect-diploes; the high-performance in In+Nb co-doped TiO_2_ ceramics has been suitably explained by Hu *et al*.^1^. However, Li *et al*. have proposed that conducting grains and resistive grain boundaries are formed in co-doped TiO_2_ (In^3+^ and Nb^5+^ doped TiO_2_) system, which leads to the internal barrier layer capacitance (IBLC) model. In IBLC model, conducting grains are separated by insulating grain boundaries, which act like capacitors. The CP behavior of materials can be explained on the basis of semiconducting grains and insulating grain boundaries^15–17^. There are other mechanisms used to explain the overall dielectric response in TiO_2_-based ceramics, including electron hopping^18^ and non-Ohmic sample-electrode contact^3^. In addition, following researches introduced more similar types of ceramics such as Ga+Nb and Al+Nb co-doped TiO_2_^2–4, 14^. However, both of the Ga+Nb and Al+Nb co-doped rutile TiO_2_ ceramics were also reported to show poor dielectric properties compared with the In+Nb co-doped in TiO_2_ ceramics^1, 3, 4, 13, 16, 17, 19–21^. Thus, developing more acceptor/donor for the AB co-doped rutile TiO_2_ ceramics is essential for pursuing high quality colossal permittivity ceramic materials.

In this study, dielectric materials with the composition of (Y_0.5_Nb_0.5_)_x_Ti_1−x_O_2_ (x = 0.001–0.1) ceramics were prepared by a conventional solid-state method. The microstructures, dielectric properties, and impedance spectra of these YNTO ceramics were studied, and then we obtained highly temperature and frequency stabilized ceramics (Y_0.5_Nb_0.5_)_0.02_Ti_0.98_O_2_.

## Results and Discussion

**Figure 1:**
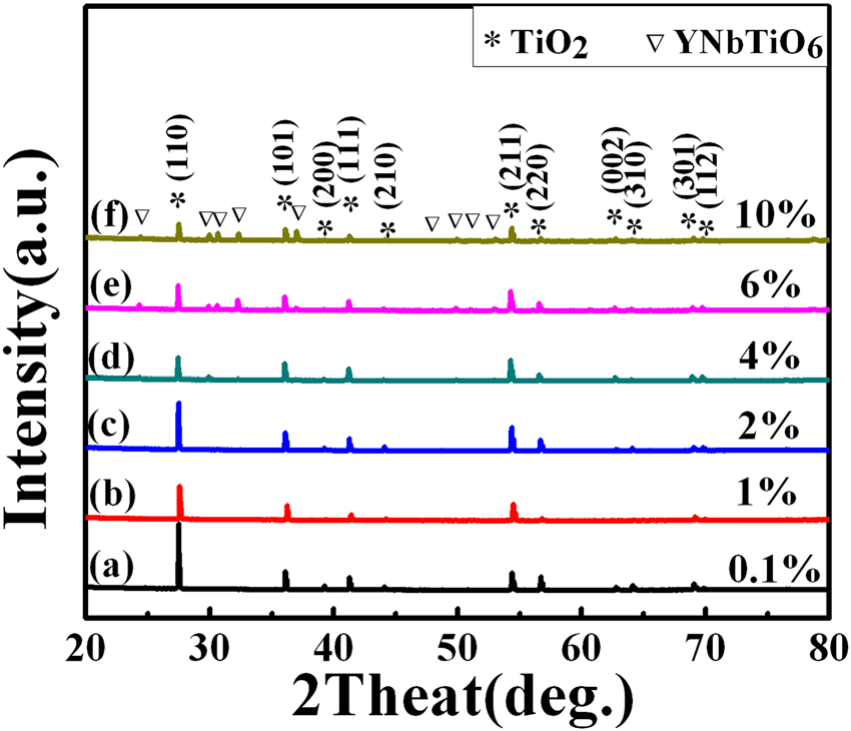
Determination of the phase structure for YNTO ceramics. (**a**)–(**f**) XRD spectra of (Y_0.5_Nb_0.5_)_x_Ti_1−x_O_2_ when x = 0.001, 0.01, 0.02, 0.04, 0.06 and 0.1.

XRD spectrums of all sintered YNTO ceramics with different co-doping concentrations are plotted together for comparison in Fig. 1. All the YNTO ceramics show pure tetragonal rutile TiO_2_ (JCPDS 21–1276) phase when x varies from 0.001 to 0.02. However, when the quantity x is further increased to 0.04, the secondary phase of YNbTiO_6_ starts to show up and is detected in 0.04, 0.06 and 0.1 samples due to the phase transformation caused by the excessive Y^3+^ and Nb^5+^ co-doping into the TiO_2_ lattice, Moreover, both the a and c values of all YNTO ceramics are comparable to those reported in the literature for the In+Nb co-doped ceramics^20^. For clearer comparison, the lattice parameters of YNTO ceramics are listed in Table 1. According to Table 1, the cell volume expands (the a and c parameters increase) when x varies from 0.001 to 0.02, as is resulted from the substitution of small Ti^4+^ (ionic radius = 60.5 pm) by the larger Nb^5+^ (ionic radius = 64 pm) and Y^3+^ (ionic radius = 90 pm) in the TiO_2_ lattice. When the quantity x is further increased to 0.04, 0.06 and 0.1, the lattice parameters of the co-doped ceramics show an abnormal tendency that can be attributed to the formation of an impurity phase.

**Table 1:** Crystal parameters of YNTO ceramics.

Composition	Lattice	Lattice	Densities		Relative
	parameter	parameter	Therotical	Measured	
					density
	a(Å)	c(Å)	g/cm^3^		
(Y_0.5_Nb_0.5_)_0.001_Ti_0.999_O_2_	4.588	2.954	4.27	3.89	91.10
(Y_0.5_Nb_0.5_)_0.01_Ti_0.99_O_2_	4.589	2.954	4.29	3.93	91.61
(Y_0.5_Nb_0.5_)_0.02_Ti_0.98_O_2_	4.591	2.956	4.30	3.94	91.63
(Y_0.5_Nb_0.5_)_0.04_Ti_0.96_O_2_	4.598	2.959	4.33	4.38	91.22
(Y_0.5_Nb_0.5_)_0.06_Ti_0.94_O_2_	4.598	2.958	4.38	3.99	91.10
(Y_0.5_Nb_0.5_)_0.10_Ti_0.90_O_2_	4.596	2.949	4.49	4.06	90.42

Crystal parameters of (Y_0.5_Nb_0.5_)_x_Ti_1−x_O_2_ ceramics when x = 0.001, 0.01, 0.02, 0.04, 0.06 and 0.1.

**Figure 2:**
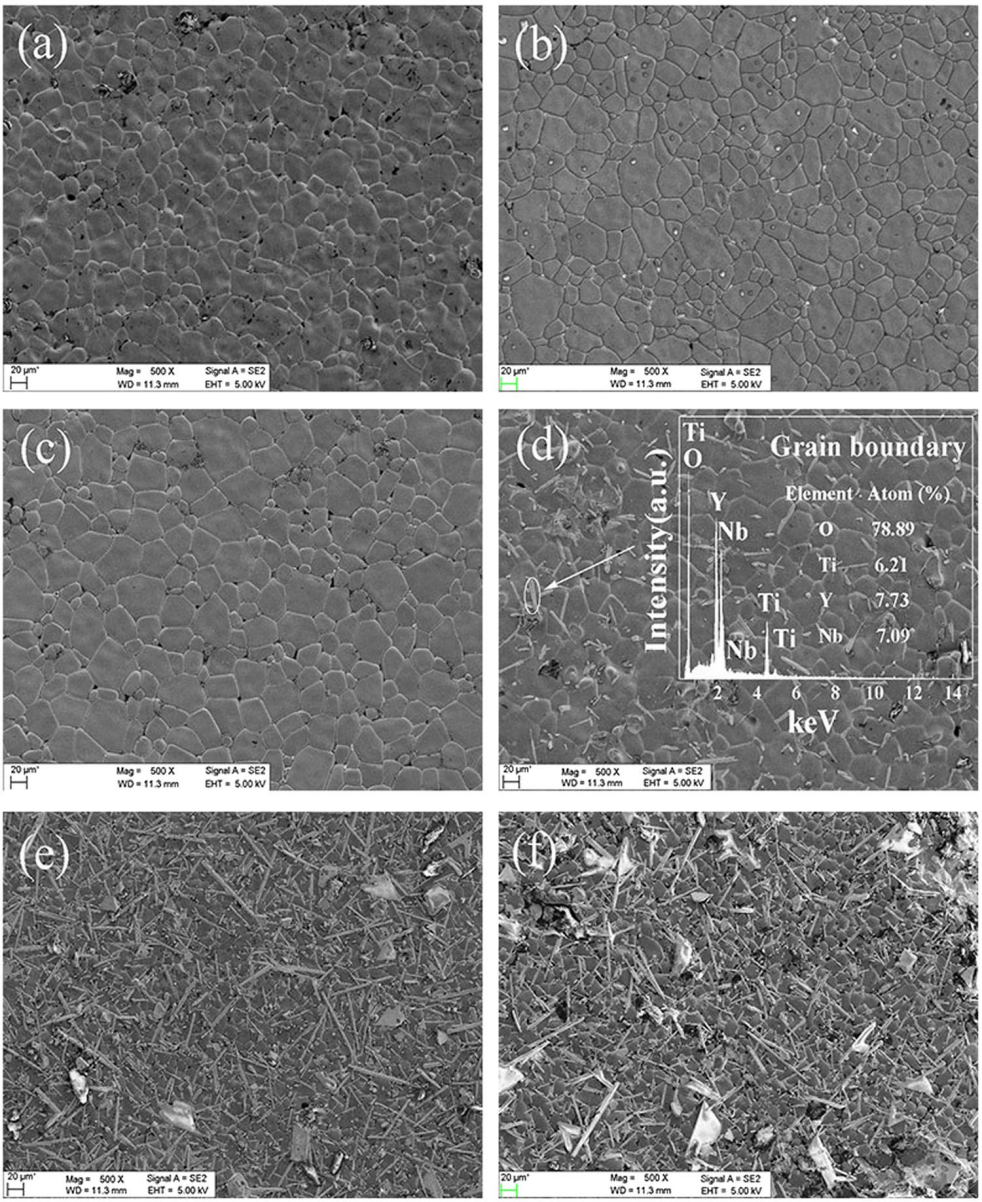
The SEM images for YNTO ceramics. (**a**)–(**f**) SEM images of (Y_0.5_Nb_0.5_)_x_Ti_1−x_O_2_ when x = 0.001, 0.01, 0.02, 0.04, 0.06 and 0.1; the inset in (**d**) shows the energy dispersion spectrum of pointed region when x = 0.04.

SEM images reveal that the grain sizes of all the sintered YNTO ceramics increase and then decrease versus x in Fig. 2. The grain size of all YNTO ceramics reaches the largest value when x = 0.02, which is consistent with Nb, In and Al, Sb co-doped TiO_2_ ceramics^1, 22^. For 0.04 sample, the EDS results of the as pointed region confirm the elemental ratio of Y:Nb:Ti is close to 1:1:1 (Y: Nb: Ti = 6.2:7.73:7.09), which is in accordance with the XRD results for the YNbTiO_6_ phase (the elemental ratio in another zone is also close to 1:1:1, as is shown in Fig. S1). In addition, when further enlarge the co-doping ratio of Y and Nb over 0.04, the grain boundaries start to restrain instead of expanding which should be ascribed to the formation of secondary phase, leading to the retardation of grain growth. As shown in Table 1, the relative densities increase to the maximum value at x = 0.02 and then decrease when co-doping ratio over 0.04, which is commensurate with the XRD and SEM data, demonstrating the solidifying benefit from the Y and Nb co-doping. Therefore, the Y and Nb co-doping profit the YNTO ceramics in enlarged cell volume, broadened grain boundary and more compact density, which would theoretically enhance the dielectric properties. To figure out this prediction, the stabilities of dielectric constant and loss are tested versus a wide range of temperature (20 to 180 °C) and frequency (10^2^–10^6^ Hz).

**Figure 3:**
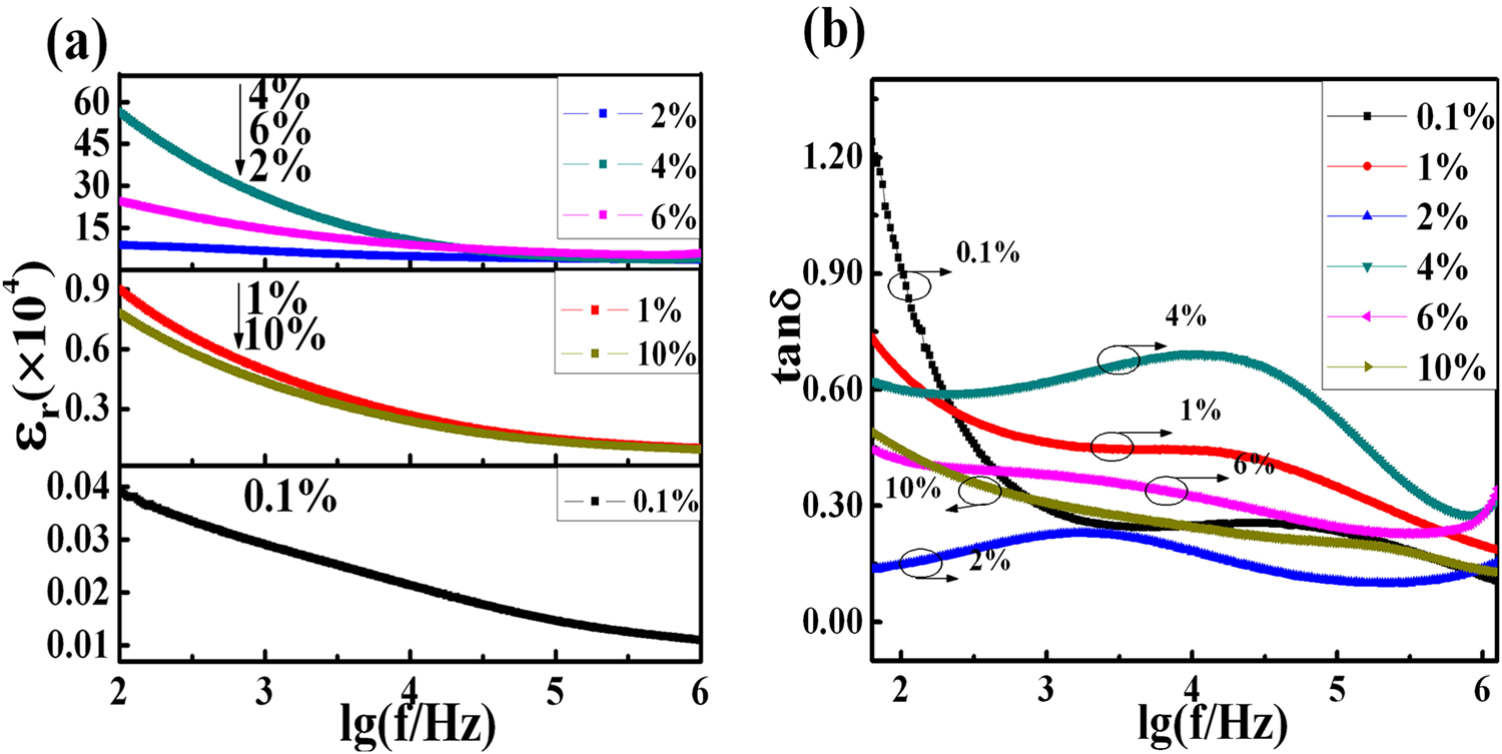
The dielectric behavior of YNTO ceramics. The effects of frequency on (**a**) dielectric constant and (**b**) dielectric loss at room temperature.

In general, all the co-doped YNTO ceramics exhibit relatively higher dielectric constant than the pure TiO_2_, except for the co-doping as low as 0.001 which shows comparable ε_r_ with the pristine^15^. The YNTO ceramics show the similar behavior of dielectric constant change versus frequency at room temperature, i.e. the value of ε_r_ drops with the increase of frequency, as shown in Fig. 3(a). The observed high dielectric constant in the low-frequency range is caused by the space polarization as was also demonstrated in the NiO-based system^6^. Fortunately, the dielectric constant increases by several magnitudes when the co-doping concentration is higher than 0.01. Regarding the co-doping content, 0.01 and 0.1 reach the similar enhancement effect indicating the negative effect of excessive co-doping. While 0.02, 0.04 and 0.06 co-doping boost the dielectric constant to almost two orders higher than 0.01 and 0.1, demonstrating the proper amount of Y and Nb co-doping region. The optimal co-doping level is determined to be 0.02, where the YNTO composite remain a rather stable and high dielectric constant (ε_r_ ~ 6.55 × 10^4^ at 1 kHz) over a wide frequency range from 10^2^ Hz to 10^6^ Hz. Unfortunately, this enhancement disappears when co-doping concentration is further increased over 0.04 where secondary phase starts to form as was verified in the XRD spectrum analysis. Hence, the secondary phase formation will decrease the dielectric constant. Though the highest ε_r_ value (~2.58 × 10^5^ at 1 kHz) is obtained for the YNTO ceramic when x = 0.04, it decreases quickly with the increasing of frequency, which is unsatisfying. Another important parameter for dielectric property is the dielectric loss along with frequency increase. As shown in Fig. 3(b), the curve of tan δ versus frequency shows a dissipation peak, and the increase of dielectric loss at low frequency might due to the direct current conduction. Meanwhile, the dielectric loss varies with x content, and the lowest value at 1 kHz is observed in (Y_0.5_Nb_0.5_)_0.02_Ti_0.98_O_2_ (tan δ = 0.29, 0.46, 0.22, 0.62, 0.38 and 0.31 when x = 0.001, 0.01, 0.02, 0.04, 0.06 and 0.1, respectively). Furthermore, both the 0.02 and 0.06 co-doped YNTO ceramics exhibit quite stable dielectric loss under the entire tested frequency range while the 0.02 co-doped ceramic exhibits relatively lower tan δ value.

**Figure 4:**
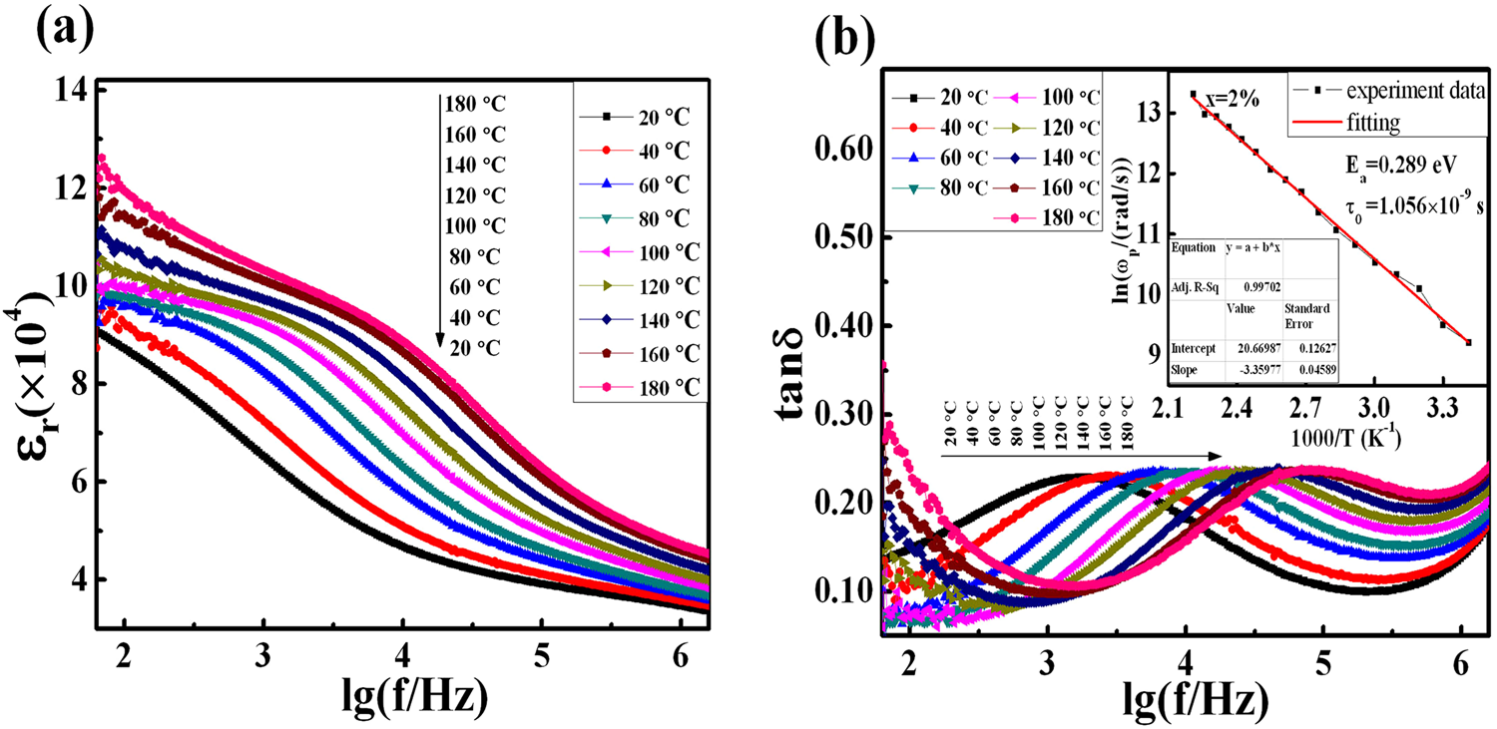
The dielectric behavior of YNTO ceramics. The effects of frequency on (**a**) dielectric constant and (**b**) dielectric loss of (Y_0.5_Nb_0.5_)_0.02_Ti_0.98_O_2_ sample under divergent temperatures; the inset in (**b**) shows the Arrhenius fitting plot of the tan δ relaxation values.

To figure out the stability of the optimized (Y_0.5_Nb_0.5_)_0.02_Ti_0.98_O_2_ ceramic as a function of frequency at different temperatures, its dielectric constant (Fig. 4(a)) and dielectric loss (Fig. 4(b)) are examined from 20 °C to 180 °C. The overall value of ε_r_ is above 10^4^ and it enlarges rapidly as a function of temperature, which can be attributed to the rise of conductivity at higher temperature^5, 21, 23, 24^. The existence of the plateau of the dielectric constant at low-frequency range demonstrates a high frequency-independence property in accordance with previous analysis. Then, the dielectric constant decreases to another plateau at higher frequencies. The increasing of dielectric constant as a function of temperature could lead to the variation of dielectric loss. Regarding the dielectric loss, the dissipation peak appears at medium frequency range (~10^3^–10^5^ Hz) with the highest value which is typical for CP co-doped ceramics^10, 17^. The relaxation peak could be fitted well with Debye relaxation model, as shown in Fig. S2. Additionally, Fig. 4(b) depicts that there should be a dissipation peak shifting to higher frequency with the increasing of temperature, which is in correspondence to the Debye relaxation^1, 3, 9, 25–27^. The dielectric relaxation time (τ) could be calculated with the extreme value relation

1 $$\mathrm{ln}({{\rm{\omega }}}_{{\rm{p}}}{\rm{\tau }})=0,$$

where circular frequency ω_p_ equals to 2πf_p_ and f_p_ is the characteristic frequency at the peak of tan δ. The fast increase of f_p_ indicates the decrease of τ with temperature increase, which is due to the thermally excited relaxation process^1, 3, 7, 24^. The activation energies required for these relaxations can be calculated with the Arrhenius law as

2 $${\rm{\tau }}={{\rm{\tau }}}_{{\rm{0}}}\exp (\frac{{{\rm{E}}}_{{\rm{a}}}}{{{\rm{K}}}_{{\rm{B}}}{\rm{T}}}),$$

where τ_0_ is the pre-exponential factor, E_a_ is the activation energy for the relaxation, K_B_ is the Boltzmann constant, and T is the temperature corresponding to the peak of tan δ. So the equation (2) can be derived as

3 $$\mathrm{ln}\,{{\rm{\omega }}}_{{\rm{p}}}=-\frac{{{\rm{E}}}_{{\rm{a}}}}{{{\rm{K}}}_{{\rm{B}}}{\rm{T}}}-\,\mathrm{ln}\,{{\rm{\tau }}}_{{\rm{0}}}$$

A linear regression of ln(ω_p_) versus 1/T fits the data quite well, as is shown in the inset of Fig. 4(b), indicating a non-polaron type relaxation all YNTO ceramics due to a hopping motion of localized carriers^21^. The values of τ_0_ and E_a_ are calculated to be 1.056 × 10^−9^ s and 0.289 eV, and the value of E_a_ is close to the activation energies for Nb-doped rutile TiO_2_^15^. Thus the dielectric properties of the Y and Nb co-doped TiO_2_ ceramics possess high temperature stability.

**Figure 5:**
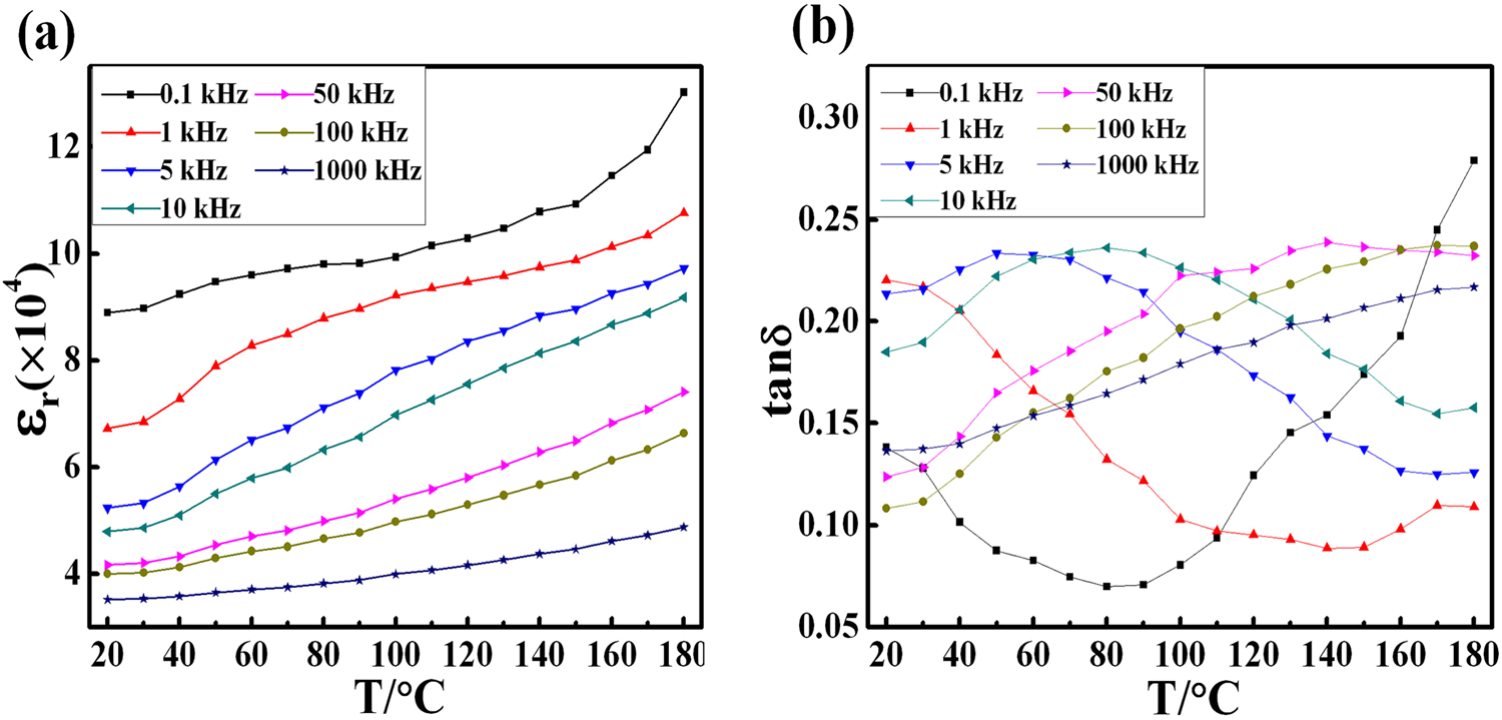
The dielectric behavior of YNTO ceramics. The effects of temperature on (**a**) dielectric constant and (**b**) dielectric loss of (Y_0.5_Nb_0.5_)_0.02_Ti_0.98_O_2_ sample under varied frequencies.

To better understand the stability of dielectric constant and dielectric loss as a function of temperature at different frequencies, the values of ε_r_ and tan δ of x = 0.02 are plotted in Fig. 5. It can be seen that the ε_r_ value increases slightly with temperature increase, indicating the high dielectric constant of Y and Nb co-doped TiO_2_ ceramics over a broad temperature range. As was detected in previous test, a broad peak of tan δ appears due to the Debye relaxor behavior. And the dielectric relaxation time would decrease with the increasing of temperature.

**Figure 6:**
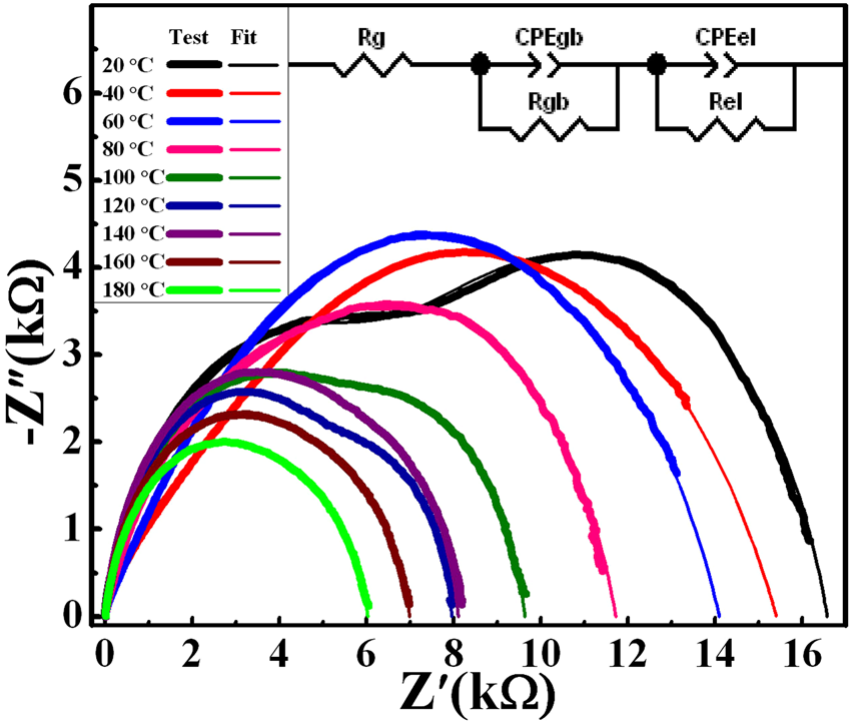
Impedance spectra for (Y_0.5_Nb_0.5_)_0.02_Ti_0.98_O_2_ ceramic at different temperatures.

Figure 6 presents the impedance spectra of the (Y_0.5_Nb_0.5_)_0.02_Ti_0.98_O_2_ sample as a function of temperature range from 20 °C to 180 °C in the frequency range of 40 Hz–10 MHz. In particular, the impedance spectra could be fitted with an appropriate equivalent circuit, as shown in the inset of Fig. 6. R_g_, R_gb_ and R_el_ are the resistance of grain, grain boundary and electrode, respectively, and CPE_gb_ and CPE_el_ is constant phase element of grain boundary and electrode, respectively. The nonzero intercept on the Z′ axis is observed at different temperatures, and it is corresponding to the grain response. The grain resistance (R_g_) values of 0.02 sample at different temperatures are ≈1–15 Ω, which are comparable to those reported in the literature^17^. In addition, the R_g_ value increases slightly with temperature, indicating that grains show conductive behavior in 0.02 sample, which is also reported in other reports^16, 17^. The left part of the arc at medium frequency corresponds to grain boundary response, and the R_gb_ decreases gradually as a function of temperature. Right part of arc at low frequency is associated with the electrode response, and the low-frequency intercept in that case gives the resistance of electrode. According to the analysis of the impedance spectrum measured at 180 °C, R_g_, R_gb_, R_el_ is about 15 Ω, 2938 Ω, and 6017 Ω, respectively. In other words, the premise of the IBLC effect (*R*_gb_ ≫ *R*_g_) is successfully established, indicating that the samples exhibit electrical inhomogeneous configuration. Based on the above discussion, the CP behavior in Y+Nb co-doped ceramic could be explained by IBLC model associated with conducting grains and insulating grain boundaries, where the electrons can move smoothly inside grains, but were accumulated in grain boundaries. Which is consistent with the prior literature^13, 15–17^, the Y and Nb co-doping in TiO_2_ ceramics enhance their dielectric properties due to the IBLC model. These satisfying results fulfill our design strategy and pave to way for more electron acceptor/donor co-doping ceramics.

In summary, (Y_0.5_Nb_0.5_)_x_Ti_1−x_O_2_ ceramics (x = 0.001, 0.01, 0.02, 0.04, 0.06 and 0.1) were obtained and their morphology and crystal structures are well examined with SEM and XRD characterizations, and the co-doping of Y+Nb resulted in an expansion of lattice parameters (the a and c values). This scarcely reported series of ceramics show stable dielectric properties even at high temperature of 180 °C. After careful tuning of the x value in (Y_0.5_Nb_0.5_)_x_Ti_1−x_O_2_ ceramics, the optimal ceramic of (Y_0.5_Nb_0.5_)_0.02_Ti_0.98_O_2_ is realized with a high value of ε_r_ (~6.55 × 10^4^) and relative low value of tan δ (~0.22). Additionally, the existence of CP behavior in sample had been explained by an internal barrier layer capacitance (IBLC) model, which consists of conducting grains and insulating grain boundaries.

## Methods

### Sample preparation

TiO_2_ (purity: 99.99%), Nb_2_O_5_ (purity: 99.9%) and Y_2_O_3_ (purity: 99.99%) were used as raw materials. The TiO_2_ and Nb_2_O_5_ were heated to dry at 200 °C for 12 h; and Y_2_O_3_ was heated at 800 °C for 2 h to decompose any carbonate. After heat treatment, the source materials were weighed immediately for subsequent weight determination. The synthesis of (Y_0.5_Nb_0.5_)_x_Ti_1−x_O_2_ (x = 0.001, 0.01, 0.02, 0.04, 0.06, 0.1) was conducted via a standard solid-state reaction method. First, the treated raw materials were mixed by ball milling in ethanol with ZrO_2_ as the medium for 10 h at 580 r/min. Second, the mixture was evaporated at 80 °C to remove the ethanol residue. Third, the resulting powder was calcined at 1100 °C for 10 h, and ground with polyvinyl acetate (PVA) solutions (5 wt. %) in an agate mortar. Finally, the resultant powder was pressed into cylindrical pellets under a pressure of 250 MPa for 5 minutes; then the pellets were sintered at 1500 °C for 10 h.

### Characterization

The crystal structures of the YNTO ceramics were identified by X-ray diffraction (XRD, Bruker D8 discover) at 40 kV and 40 mA. And their microstructures were characterized via a Field-emission scanning electron microscope (FE-SEM, Zeiss SUPRA 40). Both sides of the YNTO ceramics were coated with silver paste and heated at 650 °C to form silver electrodes; then the dielectric properties and impedance spectroscopic were determined by a precision impedance analyzer (HP, 4294 A). Moreover, the stabilities of dielectric properties for the (Y_0.5_Nb_0.5_)_0.02_Ti_0.98_O_2_ ceramic were tested in the temperature range of 20–180 °C.

## Electronic supplementary material

Dielectric properties of Y and Nb co-doped TiO2 ceramics
