# Supplementary material for: Dielectric properties of Y and Nb co-doped TiO2 ceramics
Source: Sci Rep. 2017 Aug 17;7:8517. doi: 10.1038/s41598-017-09141-0 (PMC5561205; doi:10.1038/s41598-017-09141-0)
Supplement: Supplementary file 1 — Dielectric properties of Y and Nb co-doped TiO2 ceramics [file 41598_2017_9141_MOESM1_ESM.pdf]

# Supporting Information

## Dielectric properties of Y and Nb co-doped $TiO_2$ ceramics

Xianwei Wang[1,2*], Bihui Zhang[1,2#], Linhai Xu[1,2#], Xiaoer Wang[1,2#], Yanchun Hu[1,2], Gaohang Shen[1,2], Lingyun Sun[1,2]

[1]Laboratory of Functional Materials, College of Physics and Materials Science, Henan Normal University, Xinxiang 453007, China. [2]Henan Key Laboratory of Photovoltaic Materials, Xinxiang 453007, China.

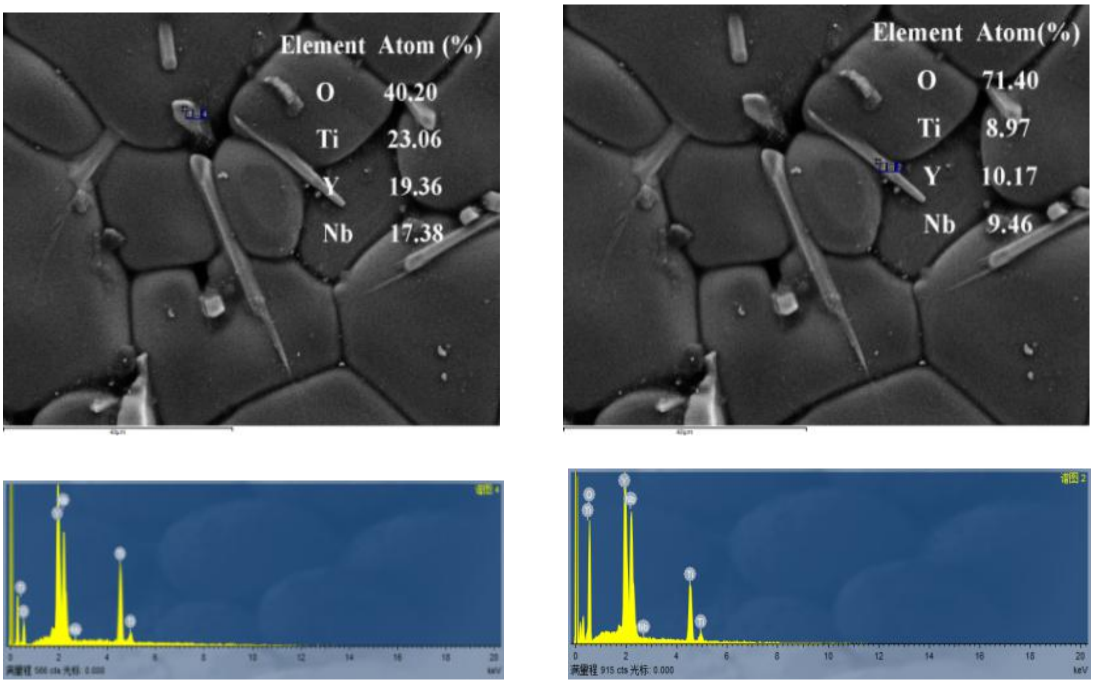


**Figure S1(a).** Energy dispersion spectrum of pointed secondary phase region when x = 0.04.

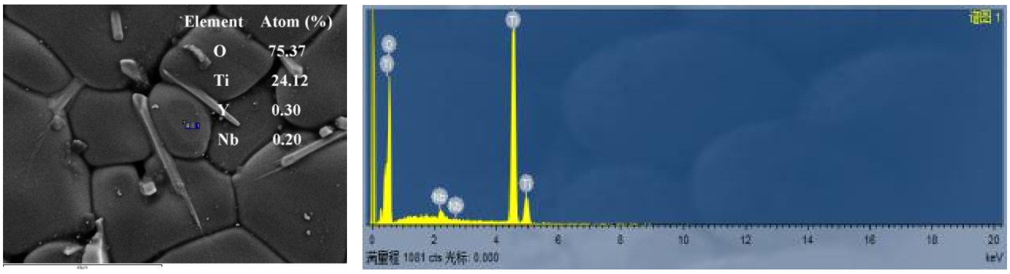


* Corresponding author
E-mail address: xwwang2000@163.com
# The three authors contributed equally to this work.

**Figure S1(b).** Energy dispersion spectrum of pointed normal grain region when x = 0.04.

From the EDS results, we can see that the composition of pointed secondary phase region is close to 1: 1: 1 (Figure S1(a)). And the major phase data shows Ti: Y: Nb =24.12: 0.3: 0.2, which is difference with initially stoichiometric (Ti: Y: Nb =96: 2: 2) due to the normal segregation (Figure S1(b)).

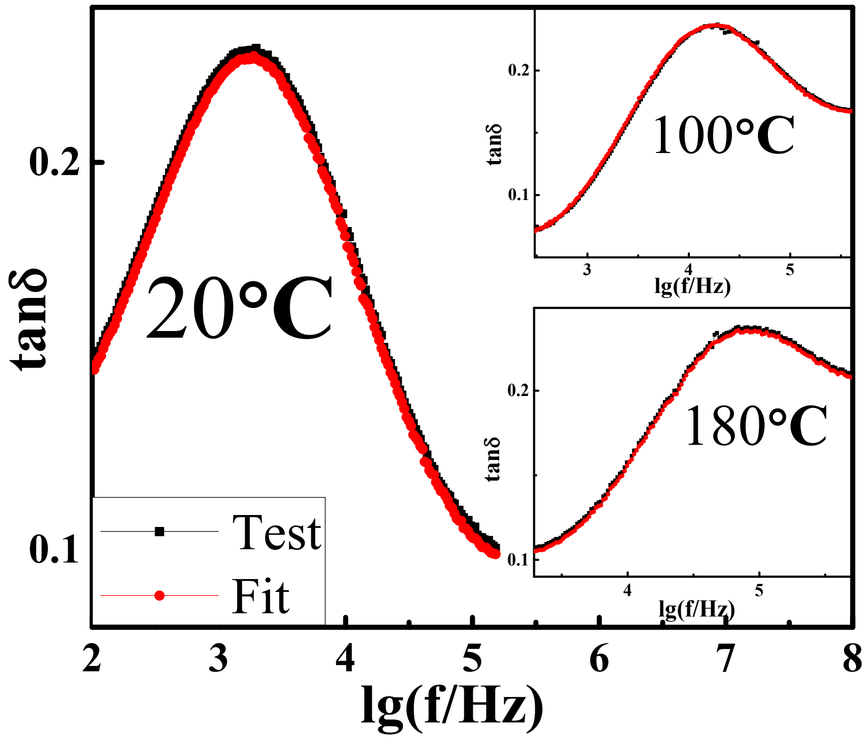


**Figure S2.** Dielectric loss relaxation peaks fitted with Debye relaxation behavior at three measurement temperatures.

The fitting results show that the relaxation peak could be fitted well with Debye relaxation behavior.
